# Comparison of the Incidence of Surgical Site Infection in Abdominal Wound Closure: Antibiotic-Coated Polyglactin 910 Suture Versus Polyglactin Suture With Local Infiltration of Antibiotic Along the Incision Line

**DOI:** 10.7759/cureus.66654

**Published:** 2024-08-11

**Authors:** Chhavindar Singh, Prakhar Pratap, Rahul Singh, Vinod K Pandey

**Affiliations:** 1 General Surgery, Motilal Nehru Medical College, Allahabad, Allahabad, IND

**Keywords:** antibiotic infiltration, polyglactin 910, triclosan, surgical site infection, laparotomy

## Abstract

Aim and objectives

The purpose of this study is to compare the incidence of surgical site infection (SSI) rates in abdominal wound closure utilizing antibacterial-coated (triclosan-coated) suture material versus conventional suture material with subcutaneous antibacterial infiltration along the incision line.

Materials and method

This prospective and comparative (randomized, non-blinded clinical trial) was conducted at the Postgraduate Department of Surgery, Swaroop Rani Nehru Hospital, associated with Motilal Nehru Medical College, Prayagraj, India. The sample size was calculated to be one hundred. The patients in Group A underwent laparotomy using polyglactin 910 coated with triclosan. The patients in Group B underwent normal suture closure and local infiltration of broad-spectrum antibiotics (1 gram of ceftriaxone in 10 ml distilled water, along with the incision).

Results

There was no discernible difference between the various groups on postoperative day (POD) 14, 21, and 30. In Group A, 100.0% of individuals had healed wound status (POD 30). Group B had healed wound status among the 96.0% of members (POD 30). Twenty percent of the people in Group A had SSI whereas 38.0% of the participants in Group B had SSI. There was no discernible difference between the two groups regarding the distribution of culture (χ² = 7.741, p = 0.127).

Conclusion

Triclosan-coated sutures are more effective than subcutaneous antibiotic infiltration along the incision line in lowering the frequency of SSI during primary laparotomy wound closure.

## Introduction

A surgical site infection (SSI) is one that arises within thirty days after surgery if no implant is left in place, or within one year if an implant is left in place. In addition to the incision, SSI can impact skin and subcutaneous tissue (superficial incisional); deep incisional soft tissue (deep incisional), such as fascia or muscle; or any other part of the body (organ/space) that was opened or moved during surgery. SSIs are caused by exogenous or endogenous bacteria that enter the surgical wound either during the surgery (primary infection) or shortly after the procedure (secondary infection) [1].

SSIs are expected to occur in 1-3.1% of all surgical procedures and are responsible for around 2.0% of all healthcare-associated infection deaths (hospital-acquired infections).

Several studies have found that the frequency of wound infection after abdominal surgery ranged from 15-25%, depending on the degree of contamination [2-5]. According to a recent study of hospital admissions after surgery in the United States, the most prevalent causes of unplanned readmission were obstruction/ileus (10.3% overall) and SSI (19.5% overall, 25.8% following colectomy/proctocolectomy) [6]. According to research conducted in India, 4-30% of surgical patients acquire SSI [7]. 

Suture materials have been the subject of much research and development in order to make them less friendly to bacterial overgrowth because they have been shown to contribute to SSI [8]. Incorporating pharmaceuticals into suture material has resulted in the creation of biologically active suture materials.

Triclosan is a broad-spectrum antiseptic agent that is particularly effective against gram-positive bacteria such as *Staphylococcus aureus, Staphylococcus epidermidis, Staphylococcus aureus, *and *Staphylococcus epidermidis*. It is less efficient when compared to gram-negative organisms. Furthermore, research indicates that triclosan has no carcinogenic, teratogenic, or genotoxic risk. Polyglactin 910 1, PDS 0, and Monocryl 3-0, three distinct triclosan-coated sutures, were compared to their uncoated equivalents [9].

The stiffness, load to failure, and strain of polyglactin 910 1 plus, PDS 0 plus, and Monocryl 3-0 plus were all measured. Variables such as loads to failure, stiffness, and strain after cylinder loading were measured and assessed for all feasible combinations. Because of their improved resistance, PDS plus sutures coated with triclosan and polyglactin 910 sutures should be utilized, according to research. The sole bioactive suture currently available is polyglactin 910 coated with triclosan, a broad-spectrum antibacterial agent effective against germs and fungi [10].

In preliminary studies, triclosan-coated suture materials were found to reduce the incidence of SSI. As a result, triclosan, a common antibacterial medication with broad-spectrum bactericidal activity against both gram-positive and gram-negative bacteria [11] was used in both in vitro and in vivo testing. SSI-causing bacteria were demonstrated to be blocked from sticking to and colonizing triclosan-coated sutures (TCS), with the effect lasting around 30 days after the sutures were implanted [12].

Several randomized clinical trials have called into question the usefulness of TCS in minimizing SSI after abdominal surgery. However, some have indicated beneficial results of TCS, while others have been unable to show statistically significant changes when compared to standard absorbable sutures [13]. In this regard, there is enough evidence to indicate that TCS material is superior to standard suture material.

The purpose of this study is to compare the incidence of SSI rates in abdominal wound closure utilizing antibacterial-coated (triclosan-coated) suture material versus conventional suture material with subcutaneous antibacterial infiltration along the incision line.

## Materials and methods

A prospective and comparative (randomized non-blinded clinical) trial was conducted at the Postgraduate Department of Surgery, Swaroop Rani Nehru Hospital associated with Motilal Nehru Medical College, Prayagraj, India. The sample size was calculated to be one hundred. The study included all patients requiring laparotomy. The study excluded patients less than eighteen years of age, patients with deep SSI or organ space SSI, and patients not willing to participate in the study. The ethical approval to conduct the study was obtained from the ethical committee of the Motilal Nehru Medical College, Prayagraj.

Study population

The study excluded patients less than eighteen years of age, patients with deep SSI or organ space SSI, and patients not willing to participate in the study. The sample size calculation was calculated using the formula N= 4PQ/LXL. The minimum sample size thus obtained was one hundred.

Source of data

The information was gathered via patient telephone calls after discharge, outpatient follow-up, and hospital records. All patients studied received abdominal closure following laparotomy. The reason for the laparotomy was not considered. Radiological tests such as chest X-rays, abdominal X-rays, and abdominal ultrasound sonography (USG) scans were completed for the patient's evaluation.

All patients in Group A who underwent laparotomy and had the muscle, fascia, rectus muscle, and subcutaneous layer closed using polyglactin 910 coated with triclosan. All patients who underwent laparotomy in Group B received normal suture closure of the muscle, rectus fascia, subcutaneous layer, and local infiltration of broad-spectrum antibiotics (1 gram of ceftriaxone in 10 ml distilled water). The aforementioned information was gathered after one month of follow-up care for these patients.

Collection of data

Before surgery, the patient's demographics, comorbidities, laparotomy rationale, setting (emergency/elective), and wound class were all gathered. The style of painting and draping, the length of the procedure, antibiotics provided during surgery, and intra-operative observations that assisted in wound categorization were all included in the intra-operative data (e.g., biliary contamination). The superficial SSI was recorded according to the CDC and Southampton wound grading system criteria.

Statistical analysis

IBM SPSS Statistics for Windows, Version 25 (Released 2017; IBM Corp., Armonk, New York, United States) was used to analyze the data using Microsoft Excel (Microsoft Corporation, Redmond, Washington, United States). Quantitative (numerical variables) data were given as mean and standard deviation, whereas qualitative (categorical variables) data were provided as frequency and percentage. The student t-test compared the two groups' mean values, while the chi-square test analyzed their frequency differences. If the p-value was less than 0.05, it was considered statistically significant.

## Results

In Group A, 34.0% of the participants were between the ages of 18 and 30. Around 8.0% of the individuals in Group A were between the ages of 31 and 40, 28.0% of the participants were between the ages of 41 and 50, and 22.0% of the participants were between the ages of 51 and 60. Of group A members, 6.0% were between the ages of 61 and 70, and 2.0% of the participants were between the ages of 71 and 80, as mentioned in Table 1.

**Table 1:** Age and gender distribution

		Group A	Group B	Total	p-value
Age range	18-30 Years	17 (34.0%)	10 (20.0%)	27 (27.0%)	0.118
	31- 40 Years	4 (8.0%)	14 (28.0%)	18 (18.0%)	
	41-50 Years	14 (28.0%)	13 (26.0%)	27 (27.0%)	
	51- 60 Years	11 (22.0%)	8 (16.0%)	19 (19.0%)	
	61-70 Years	3 (6.0%)	4 (8.0%)	7 (7.0%)	
	71-80 Years	1 (2.0%)	1 (2.0%)	2 (2.0%)	
	Mean (SD)	42.14 (15.23)	43.30 (12.47)		0.664
	Median (IQR)	44.5 (28-53.75)	44 (35.25-51.5)		
	Min – Max	19-77	21-73		
Gender	Male	28 (56.0%)	29 (58.0%)	57 (57.0%)	0.840
	Female	22 (44.0%)	21 (42.0%)	43 (43.0%)	

In Group B, 20.0% of the individuals were between the ages of 18 and 30, 28.0% of the participants were between the ages of 31 and 40, 26.0% of the participants were between the ages of 41 and 50, and 16.0% of the participants were between the ages of 51 and 60. Around 8.0% of Group B members were between the ages of 61 and 70; 2.0% of the participants were aged 71 to 80, as mentioned in Table 1.

The mean age among group A was 42.14±15.23, and group B was 43.30±12.47. Age in years was not significantly different between the two groups (W = 1186.500, p = 0.664). There was no significant difference regarding gender across the two groups (p = 0.840; χ2 = 0.041). The degree of association between the two variables was low, as mentioned in Table 1.

As mentioned in Table 2, 52.0% of the individuals in Group A had mid-line laparotomy, and 26.0% of the individuals had a Kocher incision. In Group B, 48.0% of the individuals received a mid-line laparotomy, and 28.0% of the subjects had a Kocher incision.

**Table 2:** Surgical case distribution

		Group A	Group B	Total	p-value
Type of incision	Mid-line laparotomy	26 (52.0%)	24 (48.0%)	50 (50.0%)	0.780
	Kocher incision	13 (26.0%)	14 (28.0%)	27 (27.0%)	
	McBurney’s incision	7 (14.0%)	4 (8.0%)	11 (11.0%)	
	Lower mid-line incision	2 (4.0%)	3 (6.0%)	5 (5.0%)	
	Pfannenstiel incision	2 (4.0%)	2 (4.0%)	4 (4.0%)	
	Right flank incision	0 (0.0%)	2 (4.0%)	2 (2.0%)	
	Rooftop incision	0 (0.0%)	1 (2.0%)	1 (1.0%)	
Type of surgery	Clean	6 (12.0%)	7 (14.0%)	13 (13.0%)	0.945
	Clean contaminated	30 (60.0%)	30 (60.0%)	60 (60.0%)	
	Contaminated	14 (28.0%)	13 (26.0%)	27 (27.0%)	
Type of case	Elective	32 (64.0%)	32 (64.0%)	64 (64.0%)	1.000
	Emergency	18 (36.0%)	18 (36.0%)	36 (36.0%)	

Regarding the distribution of type of incision, there was no significant difference between the two groups (χ2 = 4.135, p = 0.780). There was no significant difference between the various groups in terms of type of surgery (χ2 = 0.114, p = 0.945) and has been mentioned in Table 2.

In Group A, 64.0% of the participants seemed to be elective cases. The remaining 36.0% of the participants appeared to be emergency cases. Group B included 64.0% of participants in elective cases, and 36.0% experienced an emergency case. There was no discernible difference between the two groups (χ2 = 0.000, p = 1.000), as illustrated in Table 2.

Regarding the distribution of wound status (postoperative days two, five, seven, 14, and 21), as mentioned in Table 3, there was no significant variation between the various groups. There was no significant difference between the groups regarding the distribution of wound status (postoperative day 30). On postoperative day 30, among Group A, 100.0% of individuals had healed wound status, and among Group B, there was healed wound status among the 96.0% members. 

**Table 3:** Wound healing status

Wound status			Group A	Group B	Total	p-value
Postoperative day 2	Not healed	50 (100.0%)		50 (100.0%)	100 (100.0%)	1.000
Postoperative day 5	Not healed	50 (100.0%)		50 (100.0%)	100 (100.0%)	1.000
Postoperative day 7	Not healed	50 (100.0%)		50 (100.0%)	100 (100.0%)	1.000
Postoperative day 14	Healed	7 (14.0%)		5 (10.0%)	12 (12.0%)	0.538
	Not healed	43 (86.0%)		45 (90.0%)	88 (88.0%)	
Postoperative day 21	Healed	40 (80.0%)		41 (82.0%)	81 (81.0%)	0.799
	Not healed	10 (20.0%)		9 (18.0%)	19 (19.0%)	
Postoperative day 30	Healed	50 (100.0%)		48 (96.0%)	98 (98.0%)	0.153
	Not healed	0 (0.0%)		2 (4.0%)	2 (2.0%)	

The distribution of SSI varied significantly among the various groups (χ2 = 3.934, p = 0.047), with Group B having significantly more SSI than Group A, as illustrated in Table 4.

**Table 4:** Surgical site infection occurrence rate

Surgical site infection	Group A	Group B	Total	p-value
Present	10 (20.0%)	19 (38.0%)	29 (29.0%)	0.047
Absent	40 (80.0%)	31 (62.0%)	71 (71.0%)	
Total	50 (100.0%)	50 (100.0%)	100 (100.0%)	

As mentioned in Table 5, 80.0% of the participants in Group A had no microbial growth. Around 12.0% of the participants in Group A had *Staphylococcus aureus *growth, 4.0% of the participants in Group A had *Escherichia coli,* 2.0% of the participants in Group A had *Staphylococcus​​​​​​*​ *epidermidis, and* 2.0% of the participants in Group A had *Proteus mirabilis*. In Group B, 62.0% of the participants had no microbial growth. Around 18.0% of the participants in Group B had *Staphylococcus aureus,* 12.0% of the participants in Group B had *Escherichia coli*., 6.0% of the participants in Group B had methicillin-resistant *Staphylococcus aureus* (MRSA), and 2.0% of the participants in Group B had *Staphylococcus epidermidis*. There was no significant difference between the various groups regarding the distribution of culture (χ2 = 7.741, p = 0.127). 

**Table 5:** Result of wound culture and sensitivity MRSA: methicillin-resistant Staphylococcus aureus

Culture	Group A	Group B	Total	p-value
No growth	40 (80.0%)	31 (62.0%)	71 (71.0%)	0.127
Staphylococcus aureus	6 (12.0%)	9 (18.0%)	15 (15.0%)	
Escherichia coli	2 (4.0%)	6 (12.0%)	8 (8.0%)	
Methicillin-resistant *Staphylococcus aureus*	0 (0.0%)	3 (6.0%)	3 (3.0%)	
Staphylococcus epidermdis	1 (2.0%)	1 (2.0%)	2 (2.0%)	
Proteus mirabilis	1 (2.0%)	0 (0.0%)	1 (1.0%)	
Total	50 (100.0%)	50 (100.0%)	100 (100.0%)	

## Discussion

SSIs remain one of the most common complications of standard surgery. The most important surgery-related elements to help minimize the incidence of SSIs are thorough surgical technique, skin antisepsis, proper antibiotic prophylaxis, and the detection of wound contamination techniques. Patient-related variables such as gender, age, body mass index (BMI), comorbidities, past surgeries, and lifestyle factors are difficult to regulate [14].

According to Seiichiro Hoshino et al., triclosan, a routinely used antibiotic, shows high effectiveness against the most prevalent bacteria that cause SSIs. This broad-spectrum antibacterial agent has been used in toothpaste and soap for more than thirty years. Its method of action is thought to involve randomly disrupting the bacterial cell's membrane [15]. Galal and El-Hindawy found that polyglactin 910 coated with triclosan reduced the incidence of SSIs from 15% to 7% in prospective, randomized, double-blind research. SSI rates decreased from 10.8% in a group that used polyglactin closed sutures to 4.9% in a group that used polyglactin 910 antimicrobial sutures (polyglactin 910 Plus) [16].

In patients undergoing laparoscopic surgery, Drosdeck J et al. identified risk variables for extraction site. There were 10.3% cases of SSI reported. Lower age and greater BMI were all strongly linked to an increased risk of SSI [17]. KS Kaye et al. revealed that between the ages of 17 and 65, the risk of SSI rose by 1.1% per year (P = 0.002). Those under the age of 65 had a 1.2% reduction in the probability of SSI with each subsequent year (P =.008) [18].

There was no discernible difference between groups regarding the distribution of age in this study. (χ2 = 8.024, p = 0.118). Male or female sex is an independent risk factor for SSI depending on the operation. SJS Aghdassi et al. established characteristics that may account for these variations. Overall, male patients had considerably higher incidence rate ratios and adjusted odds ratios for SSI. Five surgical categories were used to classify the included individual operations. Male patients had considerably higher SSI rates for abdominal surgery, orthopedics, and traumatology. SSI rates for patients who underwent heart and vascular surgery were noticeably higher in female patients. Other surgical subcategories and specific operations had a range of outcomes [19]. 

In 1964, a group from the US National Research Council developed a way of classifying surgical wounds depending on the degree of microbial infection [20]. There are four types of wounds that increase the risk of SSI: clean, clean-contaminated, contaminated, and filthy. In this study, SSIs were explored depending on each of the wound states on days two, five, seven, 14, 21, and 30.

There was no discernible difference between the various groups regarding the distribution of wound status on postoperative day (two, five, seven, 14, 21, 30) in the present study. Huda F et al. [21] found SSI risk variables in patients undergoing elective laparotomy in the general surgical department of an Indian tertiary care hospital. Age, gender, smoking, comorbidities, and surgical wound class on postoperative day 30 had no significant relationship with the development of SSI. The most common bacterium identified in the culture was *Escherichia coli*.

In this study, the distribution of SSI varied significantly amongst the various groups (χ2 = 3.934, p = 0.047). Twenty percent of the people in Group A had SSI. Eighty percent of those in Group A did not get SSI. Thirty-eight percent of the participants in Group B had SSI. Sixty-two percent of those who participated in Group B had no SSI.

POD two, five, seven, 14, and 30 wound statuses were shown to be statistically insignificant when compared to the presence and absence of SSI in Group A. The most noteworthy similarity was seen on postoperative day 21.

Genta Sawada et al. investigated the efficacy of subcutaneous suction drains (SSDs) versus primary skin closure (PC) in dirty wound surgery class 4. Gender, BMI, type of incision, incision length, ASA score, steroid use, presence of diabetes mellitus, peri-operative transfusion, and surgery type did not differ between the two groups. The SSD group had a lower rate of SSI (6.6%) than the PC group (23.5%; p = 0.069). In multivariable analysis, postoperative days were determined to be a large independent risk factor for incisional SSI, whereas cultural implantation had significant preventive effects on incisional SSI (p = 0.018 and p = 0.014, respectively) [22]. 

Mawalla et al. [5] investigated the occurrence, pattern, and determinants of SSI at Bugando Medical Centre Mwanza in Tanzania. Surgical site infection (SSI) was identified in 26.0% of patients, with superficial SSI in 86.2% and deep SSI in 13.8% of patients. A positive aerobic culture was reported among 86.2% of people with clinical SSI. The most frequent organism was *Staphylococcus aureus*, which accounted for 28.6%, with MRSA accounting for 18.8%, *Escherichia coli* (25%), and *Klebsiella pneumoniae* (17.9%). ESBL producers were found in 64.3% of the *Escherichia coli* isolates and 80% of the *Klebsiella pneumoniae* isolates, respectively.

There was no discernible difference between the various groups regarding the distribution of culture (χ2 = 7.741, p = 0.127). Eighty percent of the participants in Group A had no microbial growth. Twelve percent of the participants in Group A had *Staph aureus* growth. Four percent of the participants in Group A had *E. coli*. Two percent of the participants in Group A had *Staphylococcus epidermidis*. Two percent of the participants in Group A had *Proteus mirabilis*. Sixty-two percent of the participants in Group B had no microbial growth. Eighteen percent of the participants in Group B had *Staphylococcus aureus*. Twelve percent of the participants in Group B had *E. coli*. Six percent of the participants in Group B had methicillin-resistant Staphylococcus aureus (MRSA). Two percent of the participants in Group B had *Staphylococcus epidermidis*.

E Schurtz et al. [23] studied the outcomes of mesh surgery in terms of SSIs in a cohort of forty-eight consecutive patients who had closed laparotomy wounds in the acute care surgery setting. There was no statistically significant variation in re-admission distribution throughout the study group.

There were limitations. The research was carried out at a single location, and two distinct suture materials were employed in the procedure. There is no arguing that a prospective, randomized trial is ideal, but the fact that there were so few participants in the study was a restriction. More research has to be done before it can be determined whether or not the incidence of SSIs has decreased in wounds that are dirty or contaminated.

## Conclusions

We concluded that TCS is more effective than subcutaneous antibiotic infiltration along the incision line in lowering the frequency of SSI during primary laparotomy wound closure. This moderate-quality research indicates utilizing TCS to reduce the risk of SSI, particularly in clean and contaminated surgical procedures. Although the cost of TCS is more expensive than conventional methods of subcutaneous antibiotic infiltration along the incision line, the overall cost-effectiveness considering the prevention of SSI is more with TCS, hence advised.
